# Temporal Trends and Disparities in Type 2 Diabetes Mellitus and Congestive Heart Failure Mortality in the United States From 1999–2020: A CDC WONDER Analysis

**DOI:** 10.1002/hsr2.73193

**Published:** 2026-09-07

**Authors:** Syeda Sameen Afzal, Ittiqa Khatri, Mariam Bahzad, Zeniha Fatima, Aeman Wadood, Hasibullah Aminpoor, Maryam Sajid

**Affiliations:** ^1^ Department of Medicine Dow University of Health Sciences Karachi Pakistan; ^2^ Faculty of Medicine Kabul University of Medical Sciences “Abu Ali Ibn Sina” Kabul Afghanistan; ^3^ Department of Internal Medicine Rabia Balkhi National Complex Hospital Kabul Afghanistan

**Keywords:** congestive heart failure, glycemic control, insulin resistance, SGLT 2 inhibitors, type 2 diabetes mellitus

## Abstract

**Background and Aims:**

Type 2 Diabetes Mellitus (T2DM) is a growing health burden globally, and its concurrence with Congestive Heart Failure (CHF) significantly increases morbidity and mortality. However, long‐term mortality trends remain underexplored. This study aims to assess temporal trends in mortality from comorbid T2DM and CHF in the United States (U.S.) from 1999 to 2020, with emphasis on demographic and geographical disparities.

**Methods:**

We analysed U.S. mortality data from 1999 to 2020 for adults aged ≥ 25 years with coexisting T2DM and CHF using the CDC WONDER Multiple Cause of Death database. Cases were identified using the ICD‐10 codes E11 and I50.0 for T2DM and CHF, respectively. Temporal trends in crude mortality rates (CMRs) and age‐adjusted mortality rates (AAMRs) per 100,000 population were stratified by demographic and geographic characteristics and were used to estimate the annual percent change (APC) and average annual percent change (AAPC) using Jointpoint regression.

**Results:**

Between 1999 and 2020, 292,452 deaths related to T2DM, and CHF were recorded among U.S. adults, with most deaths occurring in medical facilities. The overall AAMR increased from 4.33 in 1999 to 8.98 in 2020, with a significant rise after 2014. (APC: 7.31) AAMRs varied substantially by age, sex, race, and urbanization level. AAMRs were 27.96 among adults aged ≥ 65 years versus 0.93 among those aged 25–64 years and 7.47 among males versus 5.24 among females. Across racial groups, AAMRs were highest among American Indian/Alaska Native populations at 11.53, followed by Hispanic/Latino populations at 6.76, Black/African American populations at 6.53, non‐Hispanic White populations at 6.06, and Asian/Pacific Islander populations at 4.07. AAMRs were also higher in non‐metropolitan regions at 8.58 compared with 5.62 in metropolitan regions. Geographically, the highest mortality rate was observed in West Virginia at 11.27, with the Western region also exhibiting the highest regional mortality.

**Conclusions:**

T2DM and CHF‐related mortality have increased substantially over the past two decades, with marked increases after 2014. Increased susceptibility among certain demographic and geographical groups highlight the need for integrated strategies to combine personalized care, treatment adherence, and population‐level risk factor management to reduce mortality.

## Highlights

- Type 2 diabetes mellitus is a major risk factor for congestive heart failure.
- Mortality from type 2 diabetes with congestive heart failure nearly doubled in the U.S. from 1999 to 2020.
- Men, older adults, and American Indian/Alaska Native and Hispanic populations experienced the highest rates.
- Non‐metropolitan areas and the West and Midwest regions demonstrated consistently increased mortality.

## 1 Introduction

Type 2 Diabetes Mellitus (T2DM), a growing health burden, has become one of the major causes of mortality globally, with deaths doubling over recent decades [1]. By 2019, an estimated 437.9 million people worldwide, including 37 million Americans, were affected by T2DM [2]. In the United States (U.S.), diabetes alone accounts for an annual economic burden of approximately $413 billion, including both direct medical expenses and productivity losses [3]. Persistent hyperglycemia in T2DM culminates in a wide spectrum of long‐term complications, among these, cardiovascular disease, particularly heart failure, is the most critical, projecting direct medical costs expected to exceed $53 billion by 2030 [4, 5, 6].

Congestive heart failure (CHF), a chronic condition, has been strongly associated with T2DM [7]. The incidence of CHF approximately triples in people with diabetes as compared with non‐ diabetic populations [8]. This finding is further reinforced by a meta‐analysis, which showed that each 1% increase in HbA1c is associated with a 15% elevated risk of developing CHF [9]. This disproportionate burden is driven by overlapping pathophysiological mechanisms, mainly coronary artery disease and hyperglycemia‐induced direct myocardial injury [10].

Despite the clear recognition of T2DM as a well‐established risk factor for CHF, epidemiological studies exploring combined T2DM and CHF mortality burden remain scarce [11]. This study aims to explore temporal trends in mortality from T2DM complicated by CHF in the U.S. from 1999 to 2020, focusing on demographic and geographical disparities to guide earlier detection, refine risk, and target clinical management.

## 2 Methodology

### 2.1 Study Setting and Population

The Centers for Disease Control and Prevention's Wide‐Ranging Online Data for Epidemiologic Research (CDC WONDER) database was accessed to obtain data on patients aged 25 years or older with coexisting CHF and T2DM in the U.S. Patients were specifically identified using International Statistical Classification of Diseases and Related Health Problems‐10th Revision (ICD‐10) codes I50.0 and E11 for CHF and T2DM, respectively. We examined death certificates from the Multiple Cause of Death dataset that recorded T2DM and CHF as underlying or contributory causes. Institutional Review Board approval was exempt for this study, as it utilized anonymized publicly available data and adhered to the STROBE (Strengthening the Reporting of Observational Studies in Epidemiology) guidelines [12].

### 2.2 Data Abstraction

Data spanning 1999–2020, including demographic and regional information, were collected. The U.S. population was stratified into two age groups: younger adults (25–64 years) and older adults (≥ 65 years). Categorization was based on age, gender, race, geographical location, and place of death. The racial comparison was performed between Latinos or Hispanics, Non‐Hispanic (NH) Whites, NH Blacks or African Americans, NH American Indians or Alaska Natives, and NH Asian or Pacific Islander. The study population was categorized by the National Center for Health Statistics Urban‐Rural Scheme, with Urban areas having populations of ≥ 50,000 and < 50,000 population in rural areas [13]. The U.S. was divided into four regions: Northeast, Midwest, South, and West, as per Census Bureau definitions.

### 2.3 Statistical Analysis

We calculated crude mortality rates (CMRs) and age‐adjusted mortality rates (AAMRs) per 100,000 individuals, standardized to the 2000 U.S. population [14]. CMRs were used for age‐specific analyses, while AAMRs were used to examine overall and subgroup trends. Mortality rates were stratified by year, sex, race or ethnicity, age group, location of death, and other geographical factors, with 95% confidence intervals (CIs).

Temporal trends were assessed using Joint point Regression Program (Version 5.4.0, National Cancer Institute) [15]. Annual Percent Change (APC) with 95% CI for the AAMRs and CMRs were calculated at the identified line segments linking joint points using the Monte Carlo permutation test. In addition, the Average Annual Percent Change (AAPC) was computed to summarize the overall trend across the entire study period. APCs were considered increasing or decreasing if the slope describing the change in mortality was significantly different from zero, using a two‐tailed *t‐*test with statistical significance set at *p* < 0.05.

## 3 Results

Between 1999 and 2020, a total of 292,452 deaths were attributed to T2DM and CHF in patients aged 25 years or older. Of these, the largest proportion (35.48%) occurred in medical facilities, followed by 29.18% at the decedent's residence, 28.57% in nursing homes or long‐term care facilities, 3.01% in hospice facilities, 3.59% in other, and 0.18% at unspecified locations.

### 3.1 Annual Trends for T2DM and CHF‐Related Mortality

**Figure 1:**
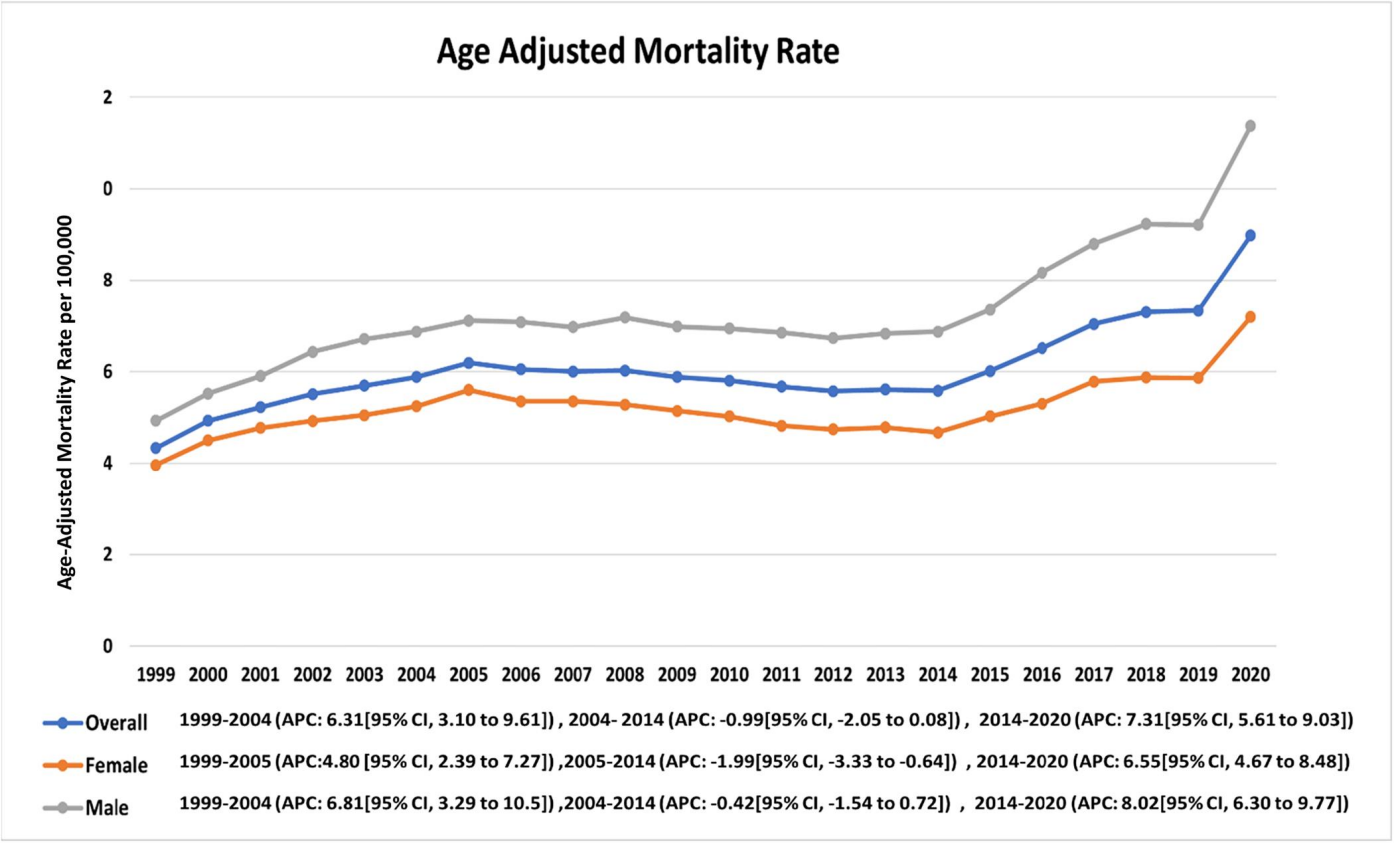
Sex stratified and overall trends in T2DM and CHF related mortality rates from 1999 to 2020.

The overall AAMR increased from 4.33 (95% CI, 4.23–4.43) in 1999 to 8.98 (95% CI, 8.86–9.09) in 2020 (Table 1). From 1999 to 2004, a steady increase was observed (APC, 6.31%; 95% CI, 3.10–9.61; *p* = 0.000753), which was followed by a non‐significant decline between 2004 and 2014 (APC, –0.99%; 95% CI, –2.05 to 0.08; *p*= 0.0664). Thereafter, a statistically significant rise from 2014 to 2020 was noted. (APC, 7.31%; 95% CI, 5.61–9.03; *p* < 0.0001) (Supplementary Table 1, Figure 1). A sensitivity analysis excluding 2020 yielded a similar temporal pattern to the primary analysis, confirming an early increase from 1999 to 2005, a subsequent decline from 2005 to 2014, marked rise from 2014 to 2017, followed by a non‐significant plateau from 2017 to 2019. This suggests that the observed post‐2014 escalation in mortality was not solely attributable to the 2020 COVID‐19 period and reflects a temporal shift that preceded the pandemic. (Supplementary Figure 1).

**Table 1:** Age‐adjusted mortality rates (AAMR) and average annual percent change (AAPC) for congestive heart failure‐ related deaths among U.S. adults aged ≥ 25 years with Type 2 Diabetes Mellitus, 1999 to 2020. *Crude Rates were used instead of AAMR.

1.	Deaths	Populations	Average AAMR (95%CI)	AAMR 1999 (95%CI)	AAMR 2020 (95%CI)	AAPC (95%CI)
Overall	292452	4473854489	6.12 (6.10–6.14)	4.33 (4.23–4.43)	8.98 (8.86–9.09)	3.04 (2.11– 3.99)
Gender						
Male	143302	2154556911	7.47 (7.43–7.51)	4.93 (4.76–5.10)	11.37 (11.18–11.57)	3.64 (2.63–4.65)
Female	149150	2319297578	5.24 (5.21–5.27)	3.96 (3.84–4.07)	7.19 (7.06–7.33)	2.32 (1.37–3.28)
Race						
NH American Indian	2713	233255	11.53 (11.07–11.98)	6.96 (5.06–9.35)	16.83 (14.82–18.83)	4.28 (1.58–7.04)
NH Asian or Pacific Islander	6793	237142712	4.07 (3.98–4.17)	1.84 (1.41–2.36)	6.61 (6.17–7.05)	5.38 (4.6121–6.16)
NH Black or African American	27247	518937524	6.53 (6.45–6.61)	4.12 (3.79–4.46)	10.29 (9.89–10.69)	3.82 (2.15–5.52)
NH White	233255	3095342890	6.06 (6.03–6.08)	4.39 (4.28–4.49)	8.63 (8.50–8.76)	2.86 (2.09–3.63)
Hispanic or Latino	21936	589350210	6.76 (6.67–6.85)	3.90 (3.45–4.35)	10.43 (10.02–10.84)	3.69 (2.37–5.02)
Census						
Northeast	37895	827193779	3.97 (3.93–4.01)	3.48 (3.29–3.67)	5.69 (5.48–5.9)	2.09 (0.86– 3.34)
Midwest	81213	969567311	7.48 (7.42–7.53)	5.81 (5.58–6.04)	10.26 (9.99–10.52)	2.15 (1.09–3.23)
South	96597	1652256217	5.63 (5.60–5.67)	4.07 (3.91– 4.23)	7.79 (7.62–7.96)	2.60 (1.71–3.50)
West	76747	1024837182	7.61 (7.56–7.66)	3.84 (3.63–4.04)	12.47 (12.19–12.76)	5.29 (4.11–6.49)
Urbanization						
Metropolitan	72670	678634169	5.62 (5.59–5.64)	3.87 (3.77– 3.97)	8.35 (8.23– 8.47)	3.28 (2.33– 4.23)
Non‐Metropolitan	219782	3795213822	8.58 (8.51–8.64)	6.19 (5.92– 6.45)	12.19 (11.86–12.52)	2.99 (1.51–4.50)
Age groups*						
Young adults (25–64) year	32829	3545377824	0.93 (0.92–0.94)	0.48 (0.44– 0.52)	1.9 (1.84–1.97)	6.55 (5.08–8.04)
Older age (65+y)	259623	928476665	27.96 (27.85–28.07)	19.93 (19.46– 20.4)	37.74 (37.23–38.25)	2.54 (1.75–3.34)

### 3.2 T2DM and CHF‐Related Mortality, Stratified by Demographics

#### 3.2.1 Age Group‐Wise Analysis

CMRs were persistently higher in older adults (Overall Crude Rate: 27.96; 95% CI: 27.85–28.07) as compared with younger individuals. (Overall Crude Rate:0.93;95% CI: 0.92 to 0.94) (Table 1). The younger population displayed a uniform increase until 2014, followed by a sharp rise thereafter (APC: 12.88; 95% CI: 10.87–14.93). Older adults demonstrated a shifting trend with an initial rise until 2005, a significant drop between 2005 and 2014 (APC: –1.83%; 95% CI: –3.05 to –0.58), and a marked surge from 2014 until the end of the study period (APC: 6.11; 95% CI: 4.40–7.85) (Supplementary Table 6, Supplementary Figure 2).

#### 3.2.2 Gender‐Wise Analysis

Throughout the study period, males consistently exhibited higher AAMRs than females, with overall rates of 7.47 (95% CI, 7.43–7.51) and 5.24 (95% CI, 5.21–5.27), respectively (Table 1). Although both sexes demonstrated increasing AAMRs, the rise was more prominent among males (AAPC, 3.64%; 95% CI, 2.63–4.65; *p*< 0.0001) in comparison to females (AAPC, 2.32%; 95% CI, 1.37–3.28; *p*< 0.0001). In females, there was a fair increase in AAMR between 1999 and 2005 (APC, 4.80%; 95% CI, 2.39–7.27; *p*= 0.0007), followed by a significant decline between 2005 and 2014 (APC, –1.99%; 95% CI, –3.33 to –0.64; *p*= 0.0072), and a sharp rebound between 2014 and 2020 (APC, 6.55%; 95% CI, 4.67–8.48; *p*< 0.0001). Males, on the other hand, exhibited significant AAMR escalations between 1999 and 2004 (APC, 6.81%; 95% CI, 3.29–10.45; *p*= 0.0009), non‐significant stabilization between 2004 and 2014 (APC, –0.42%; 95% CI, –1.54 to 0.72; *p*= 0.4421), and a conspicuous surge afterwards. (APC, 8.02%; 95% CI, 6.30–9.71; *p*< 0.0001) (Figure 1, Supplementary Table 1).

#### 3.2.3 Race‐Wise Analysis

During the study period, American Indian or Alaska Native populations exhibited the highest overall AAMR of 11.53 (95% CI:11.07 to 11.98), followed by 6.76 (95% CI:6.67– 6.85) in Hispanics or Latino, 6.53 (95% CI:6.45 to 6.61) in Blacks or African Americans 6.06 (95% CI:6.03 to 6.08) in Whites, and then 4.07 in Asian or Pacific Islander (95% CI:3.98 to 4.17) (Table 1). Across the study interval, Asian or Pacific Islanders experienced the most heightened AAMR trend (AAPC 5.38, 95% CI 4.61–6.16), followed by American Indian or Alaska Natives (AAPC = 4.28;95% CI: 1.58–7.04), Black or African American (AAPC = 3.82 95% CI 2.15–5.52) Hispanics, (AAPC = 3.69 95% CI 2.37–5.02), and Whites (AAPC = 2.86 95% CI: 2.09–3.63). Central illustration Illustration 1, 2.

**Central Illustration 1:**
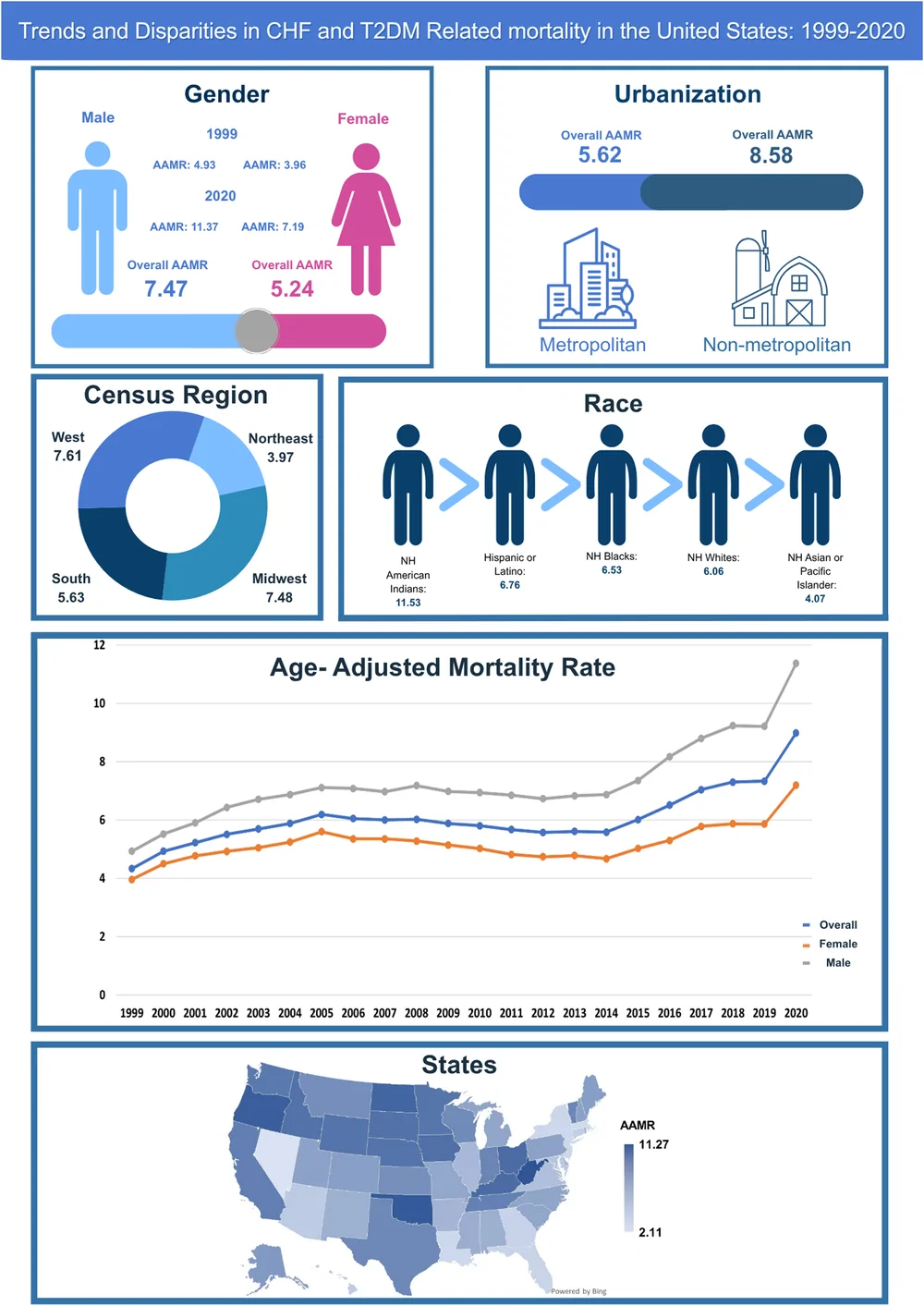
Trends and Disparities in CHF and T2DM related mortality in the United States from 1999 to 2020.

A stable rise was observed in Hispanics between 1999 and 2018 (APC: 2.61%; 95% CI: 1.94‐3.28; *p*< 0.0001) which transitioned into an upsurge from 2018 to 2020 (APC: 14.56%; 95% CI: 0.56 to 30.52; *p*= 0.041897). Amongst NH populations, AAMRs demonstrated slight variability with modest fluctuations prior to 2014 but a consistent upward trend from 2014 onwards. (Figure Illustration 1, 2, Supplementary Table 2).

**Figure 2:**
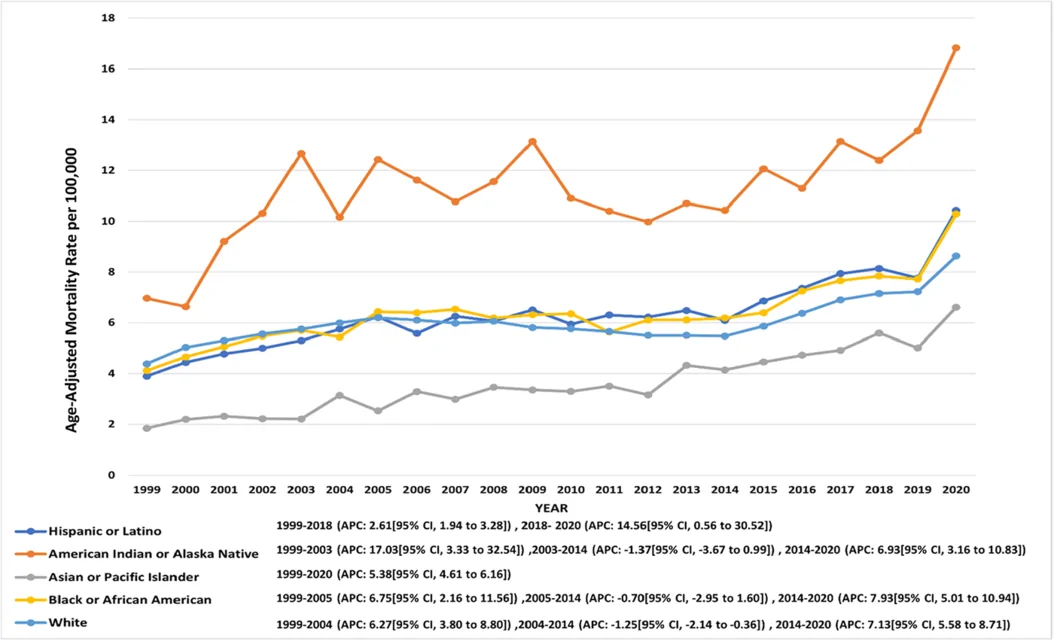
Race stratified trends in T2DM and CHF related mortality rates from 1999 to 2020.

### 3.3 T2DM and CHF‐Related Mortality, Stratified by Geography

#### 3.3.1 Urbanization‐Wise Analysis

Non‐metropolitan regions persistently reported higher AAMR (8.58; 95% CI: 8.51–8.64) than metropolitan regions (AAMR: 5.62; 95% CI: 5.59–5.64) (Table 1). In metropolitan regions, there was a uniform rise in AAMRs between 1999 and 2005 (APC: 5.19%; 95% CI: 2.66–7.78; *p*= 0.000540), followed by a subsequent decline between 2005 and 2014 (APC: –0.70%; 95% CI: –1.99–0.61; *p*= 0.270832), and a resurgence from 2014 to 2020 (APC: 7.54%; 95% CI: 5.80–9.32; *p*< 0.000001). In parallel, mortalities in non‐metropolitan regions increased from 1999 to 2005, dropped between 2005 and 2014 (APC: –2.61%; 95% CI: –3.68 –1.53; *p*= 0.000268), and rebounded with a much higher magnitude from 2014 to 2020 (APC: 8.05%; 95% CI: 6.42–9.70; *p*< 0.000001) (Supplementary Table 5, Supplementary Figure 3).

#### 3.3.2 State and Census Region‐Wise Analysis

**Figure 3:**
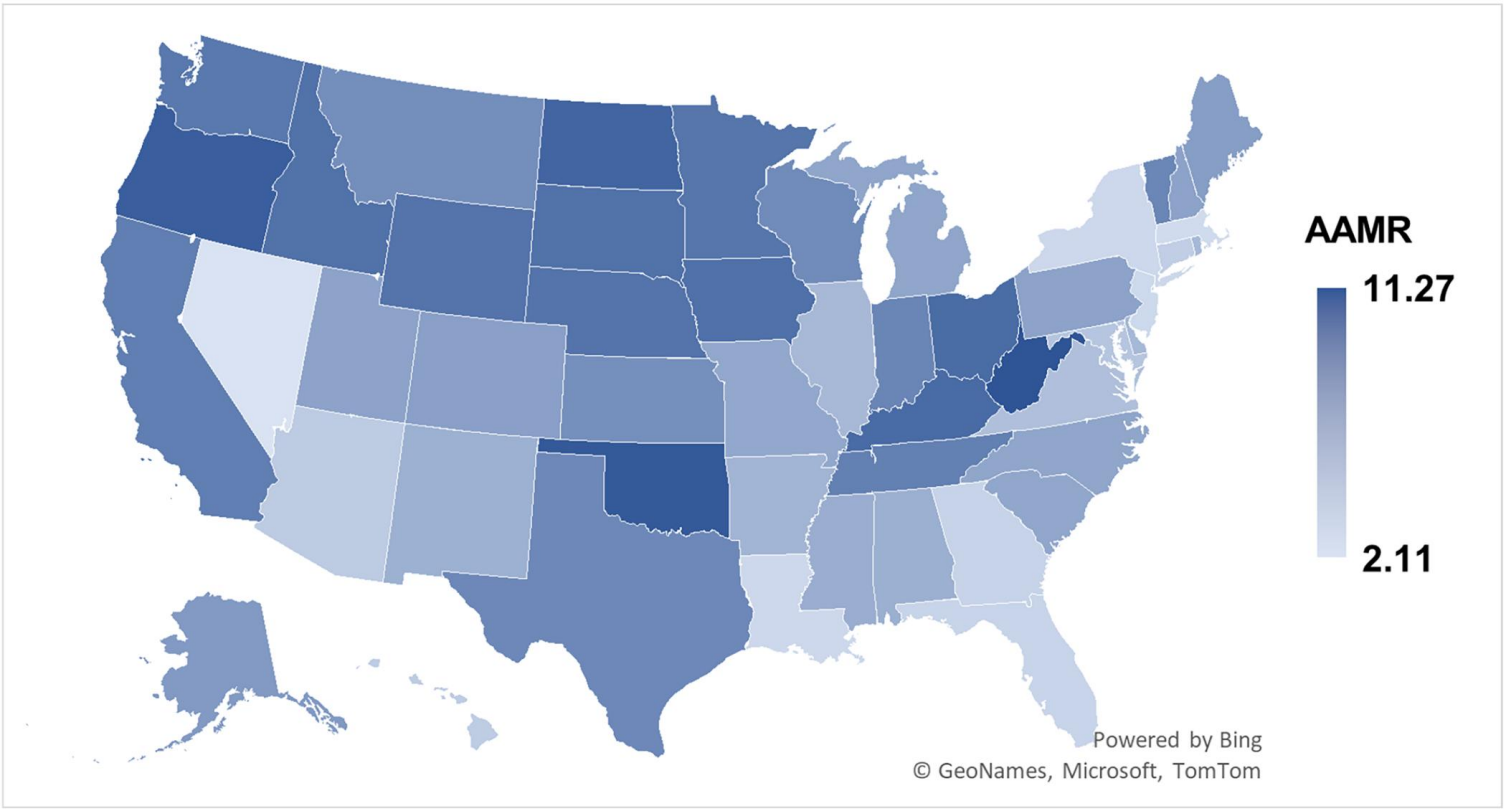
Trends in T2DM and CHF related mortality rates from 1999 to 2020 based on state wise stratification.

AAMRs varied markedly across states, from 11.27 in West Virginia to 2.11 in Nevada. States in the upper 90th percentile included West Virginia, Oklahoma, Oregon, North Dakota, and Kentucky, with AAMRs nearly threefold compared with the lower 10th percentile, which included Nevada, the District of Columbia, Massachusetts, New Jersey, and New York (Supplementary table 3, Figure 3).

Across the four US census regions, the highest mortality was observed in the West (overall AAMR: 7.61; 95% CI: 7.56–7.66) and Mid‐western regions (overall AAMR: 7.48;95% CI: 7.42–7.53), followed by South (overall AAMR: 5.63; 95% CI: 5.60–5.67), and Northeast regions (overall AAMR: 3.97; 95% CI: 3.93–4.01) (Supplementary Table 4, Supplementary Figure 4).

## 4 Discussion

This study analyzes the mortality patterns associated with T2DM and CHF from 1999 to 2020, using the CDC WONDER database. The AAMRs gradually increased until 2004, remained relatively stable until 2014, and exhibited an escalation thereafter. The upward trend was uniform across all demographic categories. Males had consistently higher AAMRs than females. American Indian or Alaska Native had the highest mortality rates among the racial groups, whereas geographically, AAMRs were highest in the Western and Midwestern regions, with non‐metropolitan areas having higher mortality rates than their metropolitan counterparts. Although the Western region exhibited the highest overall AAMRs, considerable intra‐regional variability was observed. Nevada, for instance, demonstrated one of the lowest mortality rates nationally, highlighting significant state‐level differences, with states in the top 90th percentile having mortality rates almost three times those in the bottom 10th percentile. These geographic disparities are likely multifactorial, reflecting differences in healthcare infrastructure, disease management programs, demographic composition, and regional differences in diabetes prevalence and management.

The relative stabilization and decline in AAMRs observed between 2005 and 2014 highlight improvements in cardiovascular risk management, glycemic control, and expansion of preventive public health strategies [16]. Improved risk factor profiles were consistent with enhanced healthcare access and broader insurance coverage [17, 18]. Heart failure hospitalizations and other diabetic complications also decreased substantially via better control of key risk factors such as hypertension, elevated cholesterol levels, and smoking [16, 19]. However, this trend reversed after 2014, with mortality increasing sharply through 2020. Several factors may explain this shift, including the rising prevalence of diabetes, hypertension and cardiometabolic multimorbidity in the U.S [20]. Worsening glycemic and blood pressure control at the population level and complex treatment regimens also contributed to adverse cardiovascular outcomes [21]. Increasing insulin prices and medication affordability challenges have also been associated with treatment underuse and reduced adherence [22]. Although sodium‐glucose cotransporter‐2 inhibitors (SGLT2i), which became incorporated into clinical practice in the U.S. before/around 2015, have demonstrated substantial reductions in heart failure hospitalization and cardiovascular mortality in clinical trials, their impact on the mortality trends observed in this study was likely limited [23]. The sensitivity analysis excluding 2020 demonstrated a marked increase in mortality between 2014 and 2017, followed by a non‐significant plateau from 2017 to 2019, which temporally coincided with the increasing adoption of SGLT2i in clinical practice. (Supplementary Figure 1) While this attenuation may reflect the early population‐level influence of SGLT2i and other advances in heart failure management, a causal relationship cannot be inferred from this ecological analysis [24]. Moreover, despite the increasing prescription rates, widespread uptake and routine care implementation remained limited due to high medication costs, insurance coverage restrictions and socio‐economic disparities in healthcare access [25]. Consequently, the early benefits of SGLT2i were likely insufficient to offset the increasing burden of diabetes and advanced cardiometabolic disease, resulting in persistently elevated mortality rates during the study period. Furthermore, the sharp rise in mortality, particularly observed in 2020, was likely influenced in part by the COVID‐19 pandemic. Patients with T2DM represent a high‐risk population for severe SARS‐CoV‐2 infection due to underlying metabolic and immune dysfunction [26]. In addition to direct viral effects, pandemic‐related disruptions in healthcare delivery, especially in chronic disease management, also contributed to worsening outcomes. Therefore, the observed mortality peak in 2020 should be interpreted in the context of both biological susceptibility and systemic healthcare derangement caused by the pandemic [27].

Throughout the study period, men consistently demonstrated higher AAMRs compared with women. This disparity parallels global trends, where men account for nearly 18 million more diabetes cases than women, despite women developing the disease with higher obesity rates [28, 29]. Men exhibit greater insulin resistance at lower Body Mass Index (BMI) levels, thus follow an early onset of diabetes and rapid progression to its chronic complications [30, 31]. Moreover, behavioral differences like higher rates of smoking, alcohol use, and poorer adherence to therapies and glycemic control place men at a further disadvantage [32]. By contrast, women benefit from estrogen‐mediated vascular protection during premenopausal years, which tempers early risk, but this advantage is lost after menopause, when complications accelerate, placing women at an essentially higher cardiovascular disease risk, and hence the sex gap narrows [33, 34].

Amongst the racial groups, persistently high AAMRs among NH American Indian and Alaska Native populations reflected joint burdens of early‐onset obesity and diabetes, compounded by poverty, low incomes, and underfunded health systems [35, 36, 37]. Disproportionate cardiometabolic risk, language and persistent systemic barriers contributed to elevated mortality in Hispanics and NH Black populations [38, 39, 40]. Although NH Whites exhibited comparatively lower AAMRs, the aging population sustains a considerable burden, with an amplified heart failure risk by approximately 30‐50% [41]. Sharper rises in mortality were revealed in rural communities compared with urban populations, revealing structural and healthcare barriers. Physical inactivity and higher smoking rates intensify diabetes prevalence, whereas low literacy and physician density with limited diagnostic capacity further delay recognition and treatment [42, 43, 44, 45, 46, 47, 48].

Effective management is critical to reduce morbidity and mortality associated with T2DM and CHF. The mainstay of treatment is pharmacologic therapy; SGLT2 inhibitors have significantly reduced heart failure hospitalizations and cardiovascular mortality, whereas glucagon‐like peptide‐1 (GLP‐1) receptor agonists provide complementary glycemic and vascular benefits [49, 50]. Metformin as a first‐line agent in stable disease, along with symptomatic management and heart failure‐specific therapies, is also frequently used [51, 52]. Prevention strategies focus on lifestyle modifications such as a healthy diet, regular physical activity, weight management, and smoking cessation [53]. Early identification, monitoring of risk factors, and patient education are also crucial [54, 55]. Thus, an integrated approach of combining personalized care, treatment adherence, and population‐level risk factor management strategies can enhance health and reduce mortality.

Certain limitations inherent to the study should be acknowledged. Firstly, case identification relied on ICD‐10 coding, which may exclude atypical presentations or misclassified entries. Moreover, the study defined CHF using ICD‐10 code I50.0 specifically, which improves diagnostic accuracy, but may exclude other clinically relevant heart failure subtypes. Furthermore, temporal variations in death certification and ICD‐10 coding practices during the study period may have influenced the observed mortality trends. Improvements in physician documentation and greater recognition of diabetes as a contributing cause of death may have increased the likelihood of diabetes being recorded on death certificates in later years, whereas underreporting in earlier years may have led to an underestimation of the true mortality burden, potentially exaggerating the observed increase over time [56]. Secondly, mortality data were sourced from death certificates, which are susceptible to errors in physicians' reporting and cause‐of‐death classification [57]. These data also do not account for important clinical confounders. Lastly, reliance on population‐level data does not capture individual‐level variation, restricting the broader applicability of results.

## 5 Conclusion

Mortality related to T2DM and CHF has increased substantially over the past two decades, with marked increases after 2014. Older adults consistently exhibited higher mortality, whereas the younger population demonstrated pronounced acceleration. Additionally, greater increases were observed among men, American Indian/Alaska Native, and Hispanic populations, and non‐metropolitan regions. These findings highlight the critical need for advanced strategies to counter the dual disease burden and associated disparities.

## Author Contributions

**Syeda Sameen Afzal:** data curation, software, formal analysis, methodology, investigation, writing – original draft. **Ittiqa Khatri:** visualization, writing – review and editing, investigation, conceptualization. **Mariam Bahzad:** writing – original draft, writing – review and editing, formal analysis, software. **Zeniha Fatima:** writing – original draft, conceptualization, writing – review and editing. **Aeman Wadood:** writing – review and editing, supervision, project administration. **Hasibullah Aminpoor:** writing – review and editing, supervision, project administration. **Maryam Sajid:** writing – review and editing, supervision, project administration.

## Funding

The authors have nothing to report.

## Disclosure

All authors have read and approved the final version of the manuscript. Ittiqa Khatri had full access to all data in the study and takes complete responsibility for the integrity of the data and the accuracy of the data analysis.

## Ethics Statement

No ethical approval was required for this study.

## Conflicts of Interest

The authors declare no conflicts of interest.

## Transparency Statement

Syeda Sameen Afzal affirms that this manuscript is an honest, accurate, and transparent account of the study being reported; that no important aspects of the study have been omitted; and that any discrepancies from the study as planned (and, if relevant, registered) have been explained.

## Supporting information


Supporting File

## Data Availability

The data supporting the findings of this study were obtained from the CDC WONDER online database (Centers for Disease Control and Prevention Wide‐ranging Online Data for Epidemiological Research). The datasets used and analyzed during the current study are publicly available and can be accessed at [CDC WONDER]. (https://wonder.cdc.gov).
