# Supplementary material for: Temporal Trends and Disparities in Type 2 Diabetes Mellitus and Congestive Heart Failure Mortality in the United States From 1999–2020: A CDC WONDER Analysis
Source: Health Sci Rep. 2026 Sep 7;9(9):e73193. doi: 10.1002/hsr2.73193 (PMC13548407; doi:10.1002/hsr2.73193)
Supplement: Supplementary file 1 [file HSR2-9-e73193-s001.docx]

**Supplementary Table 1.** T2DM and Congestive Heart Failure related age-adjusted mortality rates per 100,000, overall and stratified by sex in adults in the United States, from 1999 to 2020.

**Age-Adjusted Mortality Rate [95% CI]**

| Year | Overall | Male | Female |
| --- | --- | --- | --- |
| 1999 | 4.33 (4.23-4.43) | 4.93 (4.76-5.1) | 3.96 (3.84-4.07) |
| 2000 | 4.93 (4.83-5.03) | 5.52 (5.34-5.7) | 4.5 (4.38-4.63) |
| 2001 | 5.22 (5.12-5.33) | 5.9 (5.72-6.09) | 4.77 (4.65-4.9) |
| 2002 | 5.51 (5.4-5.62) | 6.43 (6.23-6.62) | 4.92 (4.79-5.05) |
| 2003 | 5.69 (5.58-5.8) | 6.71 (6.52-6.9) | 5.05 (4.91-5.18) |
| 2004 | 5.88 (5.77-5.99) | 6.87 (6.67-7.06) | 5.24 (5.11-5.37) |
| 2005 | 6.19 (6.08-6.31) | 7.11 (6.92-7.3) | 5.6 (5.46-5.73) |
| 2006 | 6.05 (5.94-6.16) | 7.08 (6.89-7.27) | 5.35 (5.22-5.49) |
| 2007 | 6 ( 5.9-6.11) | 6.97 (6.78-7.16) | 5.35 (5.22-5.48) |
| 2008 | 6.02 (5.91-6.12) | 7.18 (6.99-7.36) | 5.28 (5.15-5.41) |
| 2009 | 5.88 (5.77-5.98) | 6.98 (6.8-7.16) | 5.14 (5.02-5.27) |
| 2010 | 5.8 (5.69-5.9) | 6.94 (6.76-7.12) | 5.02 (4.9-5.15) |
| 2011 | 5.67 (5.57-5.77) | 6.85 (6.67-7.02) | 4.82 (4.7-4.95) |
| 2012 | 5.57 (5.47-5.67) | 6.73 (6.56-6.9) | 4.74 (4.62-4.86) |
| 2013 | 5.61 (5.52-5.71) | 6.83 (6.66-7) | 4.78 (4.66-4.9) |
| 2014 | 5.58 (5.48-5.68) | 6.87 (6.7-7.04) | 4.67 (4.56-4.79) |
| 2015 | 6.01 (5.91-6.11) | 7.35 (7.18-7.52) | 5.02 (4.9-5.4) |
| 2016 | 6.51 (6.41-6.61) | 8.17 (7.99-8.35) | 5.3 (5.18-5.42) |
| 2017 | 7.04 (6.94-7.15) | 8.8 (8.62-8.98) | 5.78 (5.65-5.9) |
| 2018 | 7.3 (7.19-7.4) | 9.23 (9.04-9.41) | 5.87 (5.75-6) |
| 2019 | 7.33 (7.23-7.43) | 9.21 (9.03-9.39) | 5.86 (5.74-5.98) |
| 2020 | 8.98 (8.86-9.09) | 11.37 (11.18-11.57) | 7.19 (7.06-7.33) |

**Supplementary Table 2.** T2DM and Congestive Heart Failure–related age-adjusted mortality rates per 100,000, stratified by race in adults in the United States, 1999 to 2020.

**Age-Adjusted Mortality Rate [95% CI]**

| Year | Hispanic or Latino | American Indian or Alaska Native | Asian or Pacific Islander | Black or African American | White |
| --- | --- | --- | --- | --- | --- |
| 1999 | 3.9 ( 3.45- 4.35) | 6.96 (5.06- 9.35) | 1.84 (1.41- 2.36) | 4.12( 3.79 -4.46) | 4.39 (4.28- 4.49) |
| 2000 | 4.44 ( 3.97- 4.91) | 6.63 (4.82 -8.9) | 2.19 (1.73 -2.74) | 4.66 (4.3 -5.02) | 5.03 (4.91 -5.14) |
| 2001 | 4.78 ( 4.3- 5.26) | 9.21( 7.05- 11.84) | 2.32 (1.86- 2.86) | 5.06 (4.69- 5.43) | 5.3 (5.18- 5.41) |
| 2002 | 5 ( 4.53- 5.47) | 10.31 (8.07 -12.99) | 2.22 (1.77- 2.68) | 5.47 (5.09 -5.85) | 5.56 (5.44 -5.67) |
| 2003 | 5.3 ( 4.82 5.78) | 12.67 (10.14-15.65) | 2.21 (1.78- 2.64) | 5.7 (5.32- 6.08) | 5.75 (5.63- 5.87) |
| 2004 | 5.75 (5.27- 6.24) | 10.16 (7.94 -12.82) | 3.14 (2.63 -3.65) | 5.44 (5.07- 5.82) | 5.99 (5.87 -6.11) |
| 2005 | 6.23 ( 5.74- 6.71) | 12.43 (9.97- 15.32) | 2.53 (2.1- 2.97) | 6.43 (6.03- 6.83) | 6.19 (6.07- 6.32) |
| 2006 | 5.58 (5.14- 6.03) | 11.62 (9.28 -13.95) | 3.29 (2.8 -3.78) | 6.39 (5.99 -6.78) | 6.1( 5.98 -6.22) |
| 2007 | 6.25 ( 5.79- 6.71) | 10.77 (8.6- 13.31) | 2.99 (2.54 -3.43) | 6.53 (6.14- 6.93) | 5.98( 5.87- 6.1) |
| 2008 | 6.06 ( 5.61- 6.5) | 11.56 (9.3 -13.81) | 3.46( 2.99 -3.93) | 6.18 (5.81 -6.56) | 6.05 (5.93 -6.16) |
| 2009 | 6.5 ( 6.05- 6.94) | 13.14 (10.73- 15.55) | 3.36 (2.91- 3.81) | 6.31 (5.94 6.69) | 5.81 (5.7 -5.93) |
| 2010 | 5.94 ( 5.52- 6.35) | 10.9 (8.72 -13.08) | 3.3 (2.86 -3.73) | 6.35 (5.97 -6.72) | 5.76 (5.65 -5.87) |
| 2011 | 6.3 (5.89 -6.72) | 10.4 (8.34 -12.45) | 3.51( 3.08 -3.95) | 5.63 (5.29- 5.98) | 5.65 (5.54- 5.76) |
| 2012 | 6.22 (5.82- 6.62) | 9.98 (8.01 -11.95) | 3.16 (2.77- 3.55) | 6.1 (5.74 -6.45) | 5.5 (5.39 -5.61) |
| 2013 | 6.48 (6.08- 6.87) | 10.7 (8.74 -12.66) | 4.33 (3.89- 4.78) | 6.11( 5.76- 6.45) | 5.5 (5.39- 5.6) |
| 2014 | 6.09 (5.72- 6.46) | 10.43 (8.55 -12.3) | 4.15 (3.73 -4.57) | 6.18 (5.84 -6.53) | 5.47 (5.36 -5.58) |
| 2015 | 6.86( 6.48 -7.24) | 12.06 (10.1- 14.03) | 4.46 (4.04 -4.87) | 6.39 (6.05- 6.74) | 5.86 (5.75- 5.98) |
| 2016 | 7.36 (6.98- 7.75) | 11.29 (9.43 -13.15) | 4.73 (4.31 -5.15) | 7.25 (6.88 -7.61) | 6.37 (6.25 -6.48) |
| 2017 | 7.94 (7.55 -8.33) | 13.14 (11.17- 15.11) | 4.92 (4.51- 5.33) | 7.66 (7.3- 8.02) | 6.9 (6.78- 7.02) |
| 2018 | 8.14 (7.76- 8.52) | 12.4 (10.58 -14.22) | 5.59 (5.16 -6.01) | 7.84 (7.48- 8.2) | 7.15 (7.03 -7.27) |
| 2019 | 7.76 (7.4 -8.13) | 13.57( 11.7- 15.45) | 5.01 (4.61- 5.4) | 7.72 (7.37- 8.08) | 7.22 (7.1- 7.34) |
| 2020 | 10.43( 10.02- 10.84) | 16.83 (14.82 -18.83) | 6.61 (6.17 -7.05) | 10.29 (9.89- 10.69) | 8.63( 8.5 -8.76) |

**Supplementary Table 3.** T2DM and Congestive Heart Failure related age-adjusted mortality rates per 100,000, stratified by state in adults in the United States, 1999 to 2020

**Age-Adjusted Mortality Rate [95% CI]**

| State | Age-Adjusted Mortality Rate (95% CI) |
| --- | --- |
| Alabama | 5.42 (5.25- 5.58) |
| Alaska | 6.81 (6.1 -7.52) |
| Arizona | 3.67 (3.55- 3.79) |
| Arkansas | 5.07 (4.86 -5.27) |
| California | 8.58( 8.5- 8.66) |
| Colorado | 6.4 (6.2 -6.6) |
| Connecticut | 3.45( 3.31 -3.6) |
| Delaware | 4.83 (4.47 -5.19) |
| District of Columbia | 2.15 (1.84- 2.47) |
| Florida | 3.1 (3.05 -3.16) |
| Georgia | 3.13( 3.03- 3.24) |
| Hawaii | 3.59 (3.34 -3.83) |
| Idaho | 9.42 (9.01- 9.82) |
| Illinois | 4.8 (4.7 -4.9) |
| Indiana | 8.11( 7.94- 8.29) |
| Iowa | 9.48 (9.22 -9.73) |
| Kansas | 7.48 (7.23- 7.73) |
| Kentucky | 9.8 (9.56 -10.05) |
| Louisiana | 2.83( 2.7 -2.96) |
| Maine | 6.57 (6.25 -6.89) |
| Maryland | 4 (3.86- 4.14) |
| Massachusetts | 2.62 (2.53 -2.72) |
| Michigan | 6.05 (5.93- 6.17) |
| Minnesota | 9.16 (8.95 -9.36) |
| Mississippi | 5.51 (5.29- 5.73) |
| Missouri | 5.92 (5.76 -6.07) |
| Montana | 7.5 (7.09 7.92) |
| Nebraska | 9.28 (8.94 -9.62) |
| Nevada | 2.11 (1.96- 2.27) |
| New Hampshire | 6.23 (5.89 -6.57) |
| New Jersey | 2.78( 2.69- 2.87) |
| New Mexico | 5.36( 5.1 -5.62) |
| New York | 2.81 (2.76 -2.87) |
| North Carolina | 6.07 (5.94- 6.2) |
| North Dakota | 10.2 (9.64 -10.76) |
| Ohio | 9.75 (9.61- 9.89) |
| Oklahoma | 10.97 (10.7 -11.24) |
| Oregon | 10.62 (10.36- 10.87) |
| Pennsylvania | 6.22( 6.12 -6.32) |
| Rhode Island | 4.79 (4.48- 5.1) |
| South Carolina | 5.98 (5.8 -6.16) |
| South Dakota | 9.37 (8.87- 9.87) |
| Tennessee | 8.43 (8.24 -8.61) |
| Texas | 7.92 (7.82- 8.02) |
| Utah | 6.22 (5.93 -6.52) |
| Vermont | 8.07( 7.53- 8.62) |
| Virginia | 4.36 (4.24 -4.48) |
| Washington | 8.96 (8.78- 9.15) |
| West Virginia | 11.27 (10.91 -11.63) |
| Wisconsin | 7.71 (7.53 -7.89) |
| Wyoming | 9.27 (8.6 -9.94) |

**Supplementary Table 4.** T2DM and Congestive Heart Failure–related age-adjusted mortality rates per 100,000, stratified by census region in adults in the United States, 1999 to 2020.

**Age-Adjusted Mortality Rate [95% CI]**

| Year | Northeast | Midwest | South | West |
| --- | --- | --- | --- | --- |
| 1999 | 3.48( 3.29- 3.67) | 5.81 (5.58- 6.04) | 4.07 (3.91- 4.23) | 3.84 (3.63- 4.04) |
| 2000 | 3.8 (3.61- 4) | 6.66 (6.41- 6.9) | 4.73 (4.56 -4.9) | 4.32 (4.1 -4.53) |
| 2001 | 3.95( 3.76- 4.15) | 6.95( 6.7- 7.2) | 5.01 (4.83- 5.18) | 4.76 (4.54- 4.99) |
| 2002 | 4.02 (3.82 -4.22) | 7.39 (7.14 -7.65) | 5.32 (5.14 -5.5) | 5.03 (4.8 -5.26) |
| 2003 | 4.13 (3.93- 4.330 | 7.7 (7.44- 7.96) | 5.52 (5.34- 5.7) | 5.24 (5.01- 5.47) |
| 2004 | 4.18 (3.98 -4.38) | 7.67 (7.41 -7.92) | 5.69 (5.51 -5.87) | 5.86 (5.62- 6.1) |
| 2005 | 4.21 (4.01- 4.41) | 8.14 (7.88- 8.41) | 6.05 (5.86- 6.23) | 6.12 (5.88- 6.37) |
| 2006 | 3.99 (3.8 -4.19) | 7.84 (7.59 -8.1) | 5.85 (5.67 -6.03) | 6.36 6.11 6.61) |
| 2007 | 3.6 (3.42- 3.79) | 7.72( 7.46- 7.97) | 5.76 (5.58 -5.94) | 6.79 (6.54 -7.04) |
| 2008 | 3.76 (3.57 -3.94) | 7.77 (7.52 -8.02) | 5.47 (5.3 -5.64) | 7.22 (6.97 -7.48) |
| 2009 | 3.65 (3.46 -3.83) | 7.29 (7.05 -7.53) | 5.59 (5.42- 5.76) | 6.98 (6.74 -7.23) |
| 2010 | 3.57 (3.39 -3.75) | 6.8 (6.57 -7.03) | 5.57 (5.4- 5.74) | 7.18 (6.93- 7.43) |
| 2011 | 3.5 (3.32- 3.67) | 6.86( 6.63- 7.1) | 5.15 (4.99- 5.31) | 7.28 (7.03 -7.53) |
| 2012 | 3.65 (3.47 -3.83) | 6.68 (6.46 -6.91) | 4.96 (4.8 -5.11) | 7.14 (6.9 -7.39) |
| 2013 | 3.33 (3.16- 3.5) | 6.45 (6.23- 6.67) | 5.03 (4.88- 5.19) | 7.85 (7.6 -8.1) |
| 2014 | 3.56 (3.39- 3.74) | 6.56 (6.34- 6.78) | 4.78 (4.64 -4.93) | 7.68 (7.44- 7.92) |
| 2015 | 3.69 (3.51- 3.87) | 6.87 (6.65 -7.1) | 5.14 (4.98- 5.29) | 8.55 (8.3 -8.8) |
| 2016 | 4 (3.81 -4.18) | 7.56 (7.32 -7.79) | 5.62 (5.47 -5.78) | 9.03 (8.77- 9.28) |
| 2017 | 4.13 (3.94- 4.31) | 8.28 (8.04- 8.53) | 6.12 (5.96- 6.28) | 9.88 (9.62 -10.15) |
| 2018 | 4.31 (4.12 -4.49) | 8.23 (7.99 -8.47) | 6.48( 6.32 -6.65) | 10.27( 10.01- 10.54) |
| 2019 | 4.48 (4.29- 4.67) | 8.28( 8.04- 8.52) | 6.47 (6.31- 6.63) | 10.04 (9.78 -10.3) |
| 2020 | 5.69 (5.48 -5.9) | 10.26 (9.99 -10.52) | 7.79 (7.62 -7.96) | 12.47 (12.19- 12.76) |

**Supplementary Table 5.** T2DM and Congestive Heart Failure–related age-adjusted mortality rates per 100,000, stratified by urbanization in adults in the United States, 1999 to 2020.

**Age-Adjusted Mortality Rate [95% CI]**

| Year | Metropolitan | Non-Metropolitan |
| --- | --- | --- |
| 1999 | 3.87 (3.77- 3.97) | 6.19( 5.92- 6.45) |
| 2000 | 4.38 (4.27 -4.49) | 7.24 (6.95 -7.52) |
| 2001 | 4.56 (4.45- 4.67) | 8.03 (7.73- 8.32) |
| 2002 | 4.88 (4.77 -4.99) | 8.1 (7.8- 8.4) |
| 2003 | 4.98 (4.87- 5.09) | 8.75( 8.44- 9.06) |
| 2004 | 5.25 (5.14- 5.37) | 8.74 (8.44 -9.05) |
| 2005 | 5.46 (5.35 -5.58) | 9.36 (9.05- 9.68) |
| 2006 | 5.41 (5.3- 5.53) | 8.97 (8.67 -9.28) |
| 2007 | 5.43 (5.31 -5.54) | 8.58 (8.28 -8.88) |
| 2008 | 5.41( 5.3- 5.52) | 8.78 (8.48- 9.08) |
| 2009 | 5.32 (5.22 -5.43) | 8.46 (8.16- 8.75) |
| 2010 | 5.27 (5.16 -5.38) | 8.22 (7.93 -8.51) |
| 2011 | 5.18 (5.08 -5.29) | 7.9( 7.62- 8.18) |
| 2012 | 5.1( 5- 5.21) | 7.72 (7.45- 8) |
| 2013 | 5.25 (5.14 -5.35) | 7.4 (7.13- 7.66) |
| 2014 | 5.19( 5.08- 5.29) | 7.41 (7.15 -7.68) |
| 2015 | 5.6( 5.5 -5.71) | 8.02 (7.74- 8.3) |
| 2016 | 6.14( 6.03- 6.25) | 8.38( 8.1 -8.66) |
| 2017 | 6.61 (6.49 -6.72) | 9.35 (9.05- 9.64) |
| 2018 | 6.82( 6.7- 6.93) | 9.64 (9.35 -9.94) |
| 2019 | 6.71 (6.6- 6.82) | 10.27( 9.97- 10.58) |
| 2020 | 8.35 (8.23- 8.47) | 12.19 (11.86 -12.52) |

**Supplementary Table 6.** Congestive Heart Failure related Crude Rate in patients with T2DM per 100,000 stratified by Age Group in the United States from 1999 to 2020.

| Year | Young Adults- 25 to 64 years | Old Adults- 65+ years |
| --- | --- | --- |
| 1999 | 0.48 (0.44- 0.52) | 19.93 (19.46- 20.4) |
| 2000 | 0.53 (0.49 -0.56) | 22.85 (22.34 -23.35) |
| 2001 | 0.54 (0.5- 0.58) | 24.47 (23.95- 24.98) |
| 2002 | 0.63 (0.59 -0.67) | 25.76 (25.23 -26.29) |
| 2003 | 0.69 (0.64 -0.73) | 26.75 (26.22- 27.29) |
| 2004 | 0.73 (0.69 -0.77) | 27.74 (27.2 -28.28) |
| 2005 | 0.73 (0.69- 0.77) | 29.43( 28.88- 29.99) |
| 2006 | 0.81 (0.77- 0.86) | 28.66 (28.12 -29.21) |
| 2007 | 0.81 (0.77 -0.85) | 28.46 (27.92- 28.99) |
| 2008 | 0.82 (0.78 -0.86) | 28.5 (27.97 -29.03) |
| 2009 | 0.85 (0.81- 0.9) | 27.63( 27.11- 28.14) |
| 2010 | 0.84 (0.7-9 0.88) | 27.14 (26.63 -27.64) |
| 2011 | 0.82 (0.78- 0.86) | 26.65 (26.15- 27.15) |
| 2012 | 0.85 (0.8 -0.89) | 25.72 (25.24 -26.19) |
| 2013 | 0.89 (0.85- 0.94) | 25.68 (25.21- 26.15) |
| 2014 | 0.89 (0.84 -0.93) | 25.27 (24.81 -25.73) |
| 2015 | 0.98 (0.94- 1.03) | 26.75 (26.29- 27.22) |
| 2016 | 1.14 (1.09 -1.19) | 28.59 (28.12 -29.06) |
| 2017 | 1.29 (1.23- 1.34) | 30.67 (30.19- 31.15) |
| 2018 | 1.39 (1.34 -1.45) | 31.54 (31.06 -32.02) |
| 2019 | 1.46 (1.4- 1.52) | 31.18( 30.7- 31.65) |
| 2020 | 1.9 (1.84 -1.97) | 37.74 (37.23 -38.25) |

**Crude Rate (95% CI)**

**Supplementary Figure 1.** T2DM and Congestive Heart Failure related age-adjusted mortality rates per 100,000, among adults in the United States from 1999–2019: Sensitivity analysis excluding 2020 data.

**Supplementary Figure 2.** Trends in Congestive Heart Failure-related crude rate in patients with T2DM per 100,000 stratified by age group in the United States from 1999 to 2020.

**1999-2006** (APC: 7.28[95% CI, 4.65 to 9.98]) **, 2006- 2014** (APC: 0.97[95% CI, -0.79 to 2.76]) , **2014-2020** (APC: 12.88[95% CI, 10.87 to 14.93])

**1999-2005** (APC: 5.77[95% CI, 3.41 to 8.17]) **, 2005- 2014** (APC: -1.83[95% CI, -3.05 to -0.58]) , **2014-2020** (APC: 6.11[95% CI, 4.40 to 7.85])

**Supplementary Figure 3.** Trends in Congestive Heart Failure-related age adjusted mortality rates in patients with T2DM per 100,000 stratified by urbanization in the United States from 1999 to 2020.

**Supplementary Figure 4.** Trends in Congestive Heart Failure-related age adjusted mortality rates in patients with T2DM per 100,000 stratified by census region in the United States from 1999 to 2020.

**1999-2003** (APC: 4.13[95% CI, 0.55 to 7.83])**, 2003- 2013** (APC: -2.11[95% CI, -3.02 to -1.20]), **2013-2018** (APC: 4.63[95% CI, 1.45 to 7.91]), **2018-2020** (APC: 13.87[95% CI, 4.43 to 24.18])

**1999-2005** (APC: 4.61[95% CI, 1.97 to 7.32])**, 2005- 2014** (APC: -2.70[95% CI, -4.18 to -1.20]), **2014-2020** (APC: 7.32[95% CI, 5.11 to 9.57])

**1999-2005** (APC: 5.73[95% CI, 3.43 to 8.09])**, 2005- 2014** (APC: -2.58[95% CI, -3.81 to -1.34]), **2014-2020** (APC: 7.61[95% CI, 5.84 to 9.42])

**1999-2006** (APC: 7.45[95% CI, 4.76 to 10.21])**, 2006- 2014** (APC: 2.07[95% CI, 0.14 to 4.04]), **2014-2020** (APC: 7.18[95% CI, 5.21 to 9.18])
